# Usefulness of extravascular lung water assessment as a predictor of weaning from mechanical ventilation

**DOI:** 10.1186/cc14338

**Published:** 2015-03-16

**Authors:** D Zidan, A Okasha, M Megahed, A Mahrous, R Mohamed

**Affiliations:** 1 Alexandria University Hospital, Alexandria, Egypt

## Introduction

Quantitative measurement of extravascular lung water (EVLW) would be extremely useful for clinical management, both as an index of severity and to guide treatment lung ultrasonography (LU) as a tool to quantify EVLW.

## Methods

Forty mechanically ventilated patients were examined for 5 successive days after being eligible for weaning; counting B-lines (comets) in both lung fields by LU, peak airway pressure, plateau airway pressure, static compliance and ABG analysis every 6 hours. Patients were divided into two groups according to success of the weaning process: group A (weaned group), and group B (nonweaned group).

## Results

The median value of LU comets was significantly lower in Group A compared with Group B. There was a significant increase in hypoxic index in Group A compared with Group B. There was no significant difference between both groups as regards PIP, while Pplat showed a significant increase in Group B compared with Group A and Cs showed a significant decrease in Group B.

## Conclusion

LU is a useful to quantify EVLW; it is a good predictor of weaning.
